# Pore Formation in Lipid Bilayer Membranes made of Phosphatidylcholine and Cholesterol Followed by Means of Constant Current

**DOI:** 10.1007/s12013-012-9459-6

**Published:** 2012-10-27

**Authors:** Monika Naumowicz, Zbigniew Artur Figaszewski

**Affiliations:** 1 Institute of Chemistry, University of Bialystok, Al. J. Pilsudskiego 11/4, 15-443 Bialystok, Poland; 2 Laboratory of Electrochemical Power Sources, Faculty of Chemistry, University of Warsaw, Pasteur St. 1, 02-093 Warsaw, Poland

**Keywords:** Chronopotentiometry, Electroporation, Lipid bilayer, Cholesterol, Capacitance, Resistance

## Abstract

This paper describes the application of chronopotentiometry to lipid bilayer research. The experiments were performed on bilayer lipid membranes composed of phosphatidylcholine and cholesterol and formed using the painting technique. Chronopotentiometric (*U* = *f*(*t*)) measurements were used to determine the membrane capacitance, resistance, and breakdown voltage as well as pore conductance and diameter.

## Introduction

Small pores may be created in biological and artificial membranes by applying an electric pulse of sufficient amplitude and duration. In the case of low-amplitude, short-duration pulses, the poration is reversible and the pores close within milliseconds to minutes. High-amplitude and long-duration pulses result in irreversible poration and membrane breakdown [1]. Reversible electroporation is used to introduce various substances into cells and has many practical applications in gene therapy, transdermal drug delivery, and electrochemotherapy [2–4]. Irreversible electroporation may be used for beverage and water preservation [5]. Each electroporation application has an optimal set of electrical parameters [6] that must be determined empirically [7].

It is generally thought that the pores are formed by rearrangement of lipid molecules in the bilayer structure, making the planar bilayer lipid membrane (BLM) a good model for experimental and theoretical studies [8, 9]. The BLM may be considered a small section of a cell membrane. Artificial cell membrane models may be composed of a single type of lipid molecule, a mixture of several types, or even combinations of lipids and proteins. The electrical properties of lipid bilayers are dependent on composition, and membrane composition plays an important role in determining the electroporation parameters.

When a constant voltage is applied across lipid bilayer under voltage clamp conditions, the membrane breaks down after a period dependent on the transmembrane voltage [10]. A simple way to avoid membrane failure is to work under current clamp conditions [11], also known as chronopotentiometry. The current clamp method is the only technique in which electroporated membranes survive longer than 1 h and the stochastic behavior of fluctuating electropores may be observed. The method depends on a feedback mechanism decreasing the transmembrane voltage when an excessive number of pores are opened, which in turn leads to a decrease in their size and conductance and extends the average membrane lifespan [12]. By carefully selecting the current density, it is possible to generate a single, stable, and long-lived pore in the membrane [13, 14]. In a more advanced technique known as programmable chronopotentiometry, the intensity and direction of current flow is under computer control, enabling short circuiting and disconnection of the current electrodes. This technique is useful for investigating electroporation and pore resealing and permits monitoring of the membrane structure recovery [14].

The present study is part of a comprehensive effort to fully characterize changes in the electrical properties of lipid bilayers. In the series of papers comprising references [15–18], our general goal was to measure these properties using electrochemical impedance spectroscopy. This paper describes the application of chronopotentiometry to the investigation into lipid membranes composed of phosphatidylcholine (PC) and various amounts of cholesterol (Ch). The chronopotentiometric (*U* = *f*(*t*)) characteristics of the membrane depend on the current level. At low currents, no electroporation takes place and the voltage rises exponentially to a constant value. We have demonstrated that the analysis of chronopotentiometric curves at low current densities enables simple and precise estimation of the membrane capacitance and resistance. The proposed method is also rapid enough to examine bilayers whose properties are changing in time. Both the intervention of the electrical signal (negligibly small) and the disturbances in the electrical properties are visible in the response curve. This method provides the possibility of long-term observation of disturbances in the electrical properties of BLMs under conditions of constant current flow. Higher current densities result in voltage fluctuations during electroporation, which are interpreted as the result of pores continuously opening and closing in the membrane structure. The chronopotentiometric curves recorded at high current density may be used to calculate the membrane breakdown voltage as well as the pore conductance and pore diameter.

## Theory

### Membranes at Low Current Conditions

**Fig. 1:**
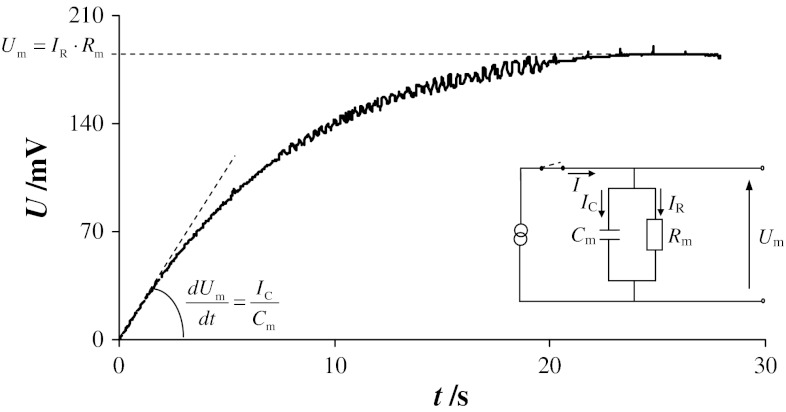
Typical chronopotentiometric curve for bilayer lipid membrane without pores (formed from solution containing phosphatidylcholine and cholesterol in a 3:7 molar ratio; current density 56 nA cm^−2^). An equivalent circuit describing the electrical properties of the bilayer is depicted in the *inset*: *C*_m_—membrane capacitance, *R*_m_—membrane resistance, *U*_m_—voltage across membrane, *I*—total current flowing through electrodes and membrane, *I*_C_—capacitance current of membrane, *I*_R_—resistance current of membrane

From an electrical point of view, a bilayer lipid membrane may be regarded as a leaky capacitor as depicted in the inset of Fig. 1. The model is composed of a membrane capacitance *C*_m_ in parallel with a membrane resistance *R*_m_. Current flowing through electrodes creates a membrane voltage *U*_m_ which reaches a constant value within a few seconds (Fig. 1).

There are two principal currents flowing through the lipid bilayer *I*: the ohmic current *I*_R_ and the charging current *I*_C_:

1 $$ I = I_{\text{R}} + I_{\text{C}} $$

where

2 $$ I_{\text{R}} = \frac{{U_{\text{m}} }}{{R_{\text{m}} }}\;{\text{and}}\;I_{\text{R}} \to I\;{\text{when}}\;t \to \;\infty $$

and

3 $$ I_{\text{C}} = C_{\text{m}} \cdot \frac{{{\text{d}}U_{\text{m}} }}{{{\text{d}}t}}\;{\text{and}}\;I_{\text{C}} \to I\;{\text{when}}\;t \to 0 $$

d*U*_m_/d*t* describes the rising part of the chronopotentiometric curves irrespective of further changes in membrane structure.

Using Eq. (3) and the known value of *I*, the membrane capacitance may be calculated from the slope of the curve. The smooth linear portion of the chronopotentiometric curve may be used to calculate the membrane resistance according to Eq. (2).

If Eq. (3) is inserted into Eq. (1), the following expression for the factor d*U*_m_/d*t* is derived:

4 $$ \frac{{{\text{d}}U_{\text{m}} }}{{{\text{d}}t}} = \frac{{I - I_{\text{R}} }}{{C_{\text{m}} }} $$

### Membranes at High Current Conditions

**Fig. 2:**
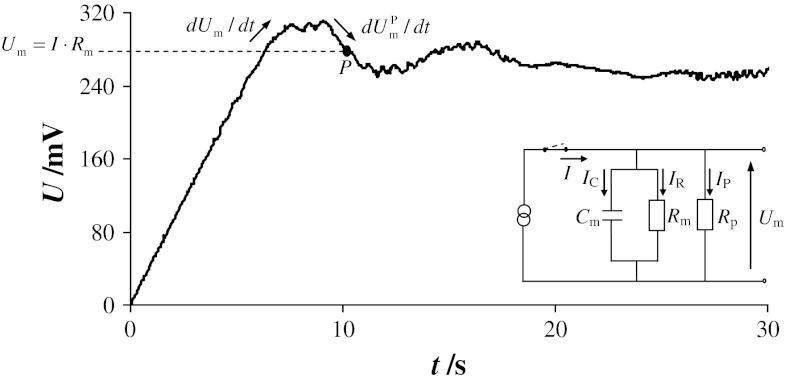
Typical chronopotentiometric curve for perforated lipid bilayer. The marked parameters were used to calculate pore conductance at the selected point *P* (membrane formed from solution containing phosphatidylcholine and cholesterol in 3:7 molar ratio; current density 100 nA cm^−2^). An equivalent circuit describing the electrical properties of the bilayer is depicted in the *inset*: *C*_m_—membrane capacitance, *R*_m_—membrane resistance, *U*_m_—voltage across membrane, *I*—total current flowing through electrodes and membrane, *I*_C_—capacitance current of membrane, *I*_R_—resistance current of membrane

Electroporation of the BLM causes the kinetics of the voltage changes across the perforated membrane ${\text{d}}U_{\text{m}}^{\text{P}} /{\text{d}}t$ (Fig. 2) to be different from an intact membrane d*U*_m_/d*t* (Fig. 1). The equivalent electrical circuit of a lipid bilayer containing pores is represented in the inset of Fig. 2. *I*_P_ denotes the current flowing through the pores, and *R*_P_ defines the pore resistance. Therefore, Eq. (1) must be supplemented with the current *I*_P._

The slope of the chronopotentiometric curve for the perforated membrane may be written as:

5 $$ \frac{{{\text{d}}U_{\text{m}}^{\text{p}} }}{{{\text{d}}t}} = \frac{{I - I_{\text{R}} - I_{\text{P}} }}{{C_{\text{m}} }} $$

When the calculations are performed using the same value of membrane voltage, Eqs. (4) and (5) give equal values for membrane capacitance, and the current flowing through the pores may be expressed using the equation:

6 $$ I_{\text{P}} = \left( {I - I_{\text{R}} } \right) \cdot \left( {1 - \frac{{{\text{d}}U_{\text{m}}^{\text{P}} /{\text{d}}t}}{{{\text{d}}U_{\text{m}} /{\text{d}}t}}} \right) $$

The pore conductance *G*_P_ may be written as a function of the bilayer voltage and the current flowing through the pores:

7 $$ G_{\text{P}} = \frac{{I_{\text{P}} }}{{U_{\text{m}} }} $$

Combining Eq. (7) with Eqs. (2) and (6), the expression for the pore conductance at any selected point *P* (Fig. 2) is obtained in the form [19]:

8 $$ G_{\text{p}} = \left( {\frac{I}{{U_{\text{m}} }} - \frac{1}{{R_{\text{m}} }}} \right) \cdot \left( {1 - \frac{{{\text{d}}U_{\text{m}}^{\text{P}} /{\text{d}}t}}{{{\text{d}}U_{\text{m}} /{\text{d}}t}}} \right) $$

Bilayer lipid membranes usually have very high resistances [9, 20], and the factor 1/*R*_m_ may be neglected in Eq. (8). An ideal lipid membrane should not conduct electricity. However, real membranes contain water molecules and ions, leading to a finite resistance value. Generally, membrane resistance is not reproducible and may vary from membrane to membrane despite the same lipids being used for membrane preparation. Hence, *R*_m_ must be estimated for each membrane used in experiments. However, the resistance of a particular membrane is usually constant until a short time before the membrane breaks, and any changes in resistance due to addition of ions, proteins, drugs, etc., may be determined with a relatively high degree of accuracy. The formation of pores also decreases the resistance.

Based on the literature, the creation of a single pore under constant current conditions is assumed [13, 14, 19, 21]. If the pore conductance is known, the pore diameter *d*_P_ may be calculated assuming that (a) the pore is cylindrical, (b) the pore is filled with the same electrolyte as the bulk electrolyte, (c) the temperature change inside the pore due to the current flow did not significantly change the pore conductance, and (d) the thickness of the membrane *L* is assumed to be 7 nm for phosphatidylcholine bilayers and 5.9 nm for phosphatidylcholine bilayers modified with cholesterol [21]:

9 $$ d_{\text{P}} = \sqrt {\frac{{G_{\text{P}} \cdot L}}{\kappa \cdot \pi }} $$

where *κ* is specific conductance of the electrolyte.

## Materials and Experimental Details

### Chemicals and Preparation of the Forming Solutions

The lipid bilayer was formed from egg lecithin (3-sn-phosphatidylcholine) produced by Sigma (61755; St. Louis, MO) and from cholesterol made by Fluka (26734; Neu-Ulm, Germany).

The lipids were dissolved in chloroform to prevent oxidation. The solvent was evaporated under a stream of argon. Dried residues were dissolved in a mixture of *n*-hexadecane and *n*-butanol (10:1 by volume). The resultant solution used to form the model membrane contained 20 mg ml^−1^ of PC or PC and Ch in the molar ratio of 7:3 (7PC–3Ch), 1:1 (1PC-1Ch), or 3:7 (3PC-7Ch). During membrane formation, the solvent mixture was removed, resulting in a membrane with the same composition as the solution. Samples were stored at 4 °C for less than 1 week. The preparation and storage methods provided reproducible electrochemical properties when samples prepared at different times were examined using chronopotentiometric method.

The electrolyte solution contained 0.1 mol dm^−3^ potassium chloride and was prepared using triple-distilled water (second distillation was made with KMnO_4_ and KOH to remove organic impurities) and KCl produced by POCh (Gliwice, Poland). The KCl was calcined to remove any organic impurities.

All solvents were chromatographic standard grade. Hexadecane was purchased from Fluka (Neu-Ulm, Germany), and chloroform and butanol were obtained from Aldrich (Milwaukee, WI).

The experiments were performed at a temperature of about 293 ± 1 K.

### Preparation of the Bilayer Membranes

Bilayer membranes were obtained as bubbles at the Teflon cap comprising a portion of the measuring vessel. The use of *n*-hexadecane as a solvent made it possible to obtain membranes with thickness and capacity values similar to those of monolayer membranes [22, 23]. The small quantity of *n*-butanol had a negligible effect on the electrical parameters of the bilayers, yet it considerably accelerated membrane formation.

Thinning of the membranes was monitored using reflected light microscopy with a high-brightness yellow LED source. The microscope and the LED were mounted on supports enabling placement of the illuminator, measuring vessel, and microscope on the optical axis. The distance of the microscope from the measuring cell could also be adjusted in order to focus on the membrane located deep within the vessel.

Bilayer formation was also monitored electrically by measuring the membrane capacitance at low frequency. The capacitance of the membranes increased with time after bilayer formation until a steady-state value was reached after approximately 5–10 min. Measurements were begun 10–15 min after the membranes turned completely black. When the capacitance stabilized, it was assumed that diffusion of solvent out of the bilayer was complete, although some hexadecane molecules might remain “dissolved” in the membrane interior.

The membrane images were captured with a color CCD camera using the WinFast PVR program (http://winfast-pvr.software.informer.com). The bilayer areas were calculated from the photographs, taking into consideration the spherical nature of the surface and using the equations provided in [24]. The area of the BLMs was about 9 × 10^−2^ cm^2^.

### Chronopotentiometry Measurements

**Fig. 3:**
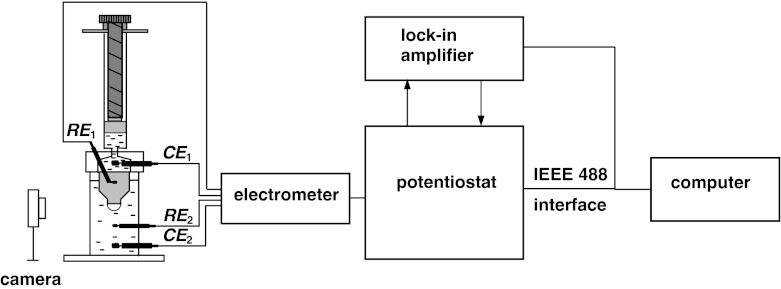
General arrangement of the measurement apparatus

The general architecture of the system used to chronopotentiometric measurements is shown in Fig. 3. The setup included a personal computer, a two-phase lock-in amplifier (EG&G, Princeton Applied Research, model 5210), and a potentiostat/galvanostat (EG&G, Princeton Applied Research, model 273A), in which a four-electrode input was applied within the self-constructed electrometer.

The electrochemical cell used for chronopotentiometric measurement with a BLM system was essentially the same as that proposed by us for impedance measurements [16] and was placed in a Faraday cage during the measurement in order to decrease the background noise. The electrochemical cell contained two identical reversible silver–silver chloride electrodes (RE_1_ and RE_2_) and two identical current platinum electrodes (CE_1_ and CE_2_). The four-electrode potentiostat assured passage of current between the two platinum electrodes in such a manner as to hold constant the amplitude of voltage between the two reversible electrodes and measured the intensity and phase of current in the circuit CE_1_–CE_2_. The use of the four-electrode system in the studies of electric phenomena occurring in membranes makes it possible to considerably reduce the errors caused by electrode and electrolyte impedance [25, 26].

Constant current chronopotentiometric measurements were taken using PowerSTEP software (part of the PowerSuite package, EG&G Princeton Applied Research). The data were processed and analyzed with the computer program Excel (Microsoft, Redmond, WA).

## Results

### Chronopotentiometric Characteristics of Bilayer Lipid Membranes

The chronopotentiometric characteristics (*U* = *f*(*t*), membrane voltage as a function of time) of pure, and cholesterol-modified phosphatidylcholine membranes were recorded under constant current conditions. Current flow through bilayers causes membrane charging, with the rate of charging dependent on the capacitance current. As a result, the membrane voltage increases with time.

The chronopotentiometric curves were recorded at several DC values. Some measurements on the same membrane were obtained in several stages using increasing current values. Other measurements were conducted at the same current until irreversible breakdown occurred, providing us with relatively long-term monitoring results. The first recorded curve was always used when comparing experimental results. The mean values of all parameters (electrical capacitance, electrical resistance, pore conductance, pore diameter, and breakdown voltage) were obtained on the basis of chronopotentiometric curves recorded for six lipid bilayers.

**Fig. 4:**
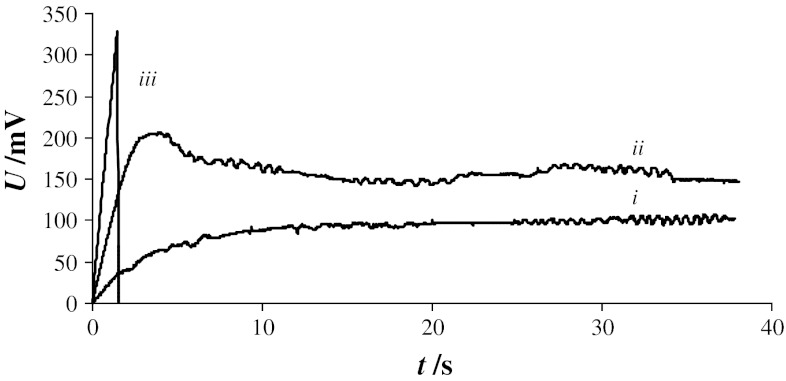
Example of chronopotentiometric curves for membrane made from phosphatidylcholine and cholesterol (1:1 molar ratio) at various constant current densities: *i*—11 nA cm^−2^, *ii*—100 nA cm^−2^, *iii*—233 nA cm^−2^

Figure 4 contains typical chronopotentiometric curves recorded in a PC–Ch system (1:1 molar ratio) at various current densities: *i*—11 nA cm^−2^, *ii*—100 nA cm^−2^, and *iii*—233 nA cm^−2^. The shapes of these curves are strongly dependent on the current. At low current densities (Fig. 4, curve *i*), no electroporation took place and the membrane voltage increased exponentially to a constant value described by Ohm’s law. Higher current densities (Fig. 4, curve *ii*) caused a faster increase in membrane voltage, followed by a sudden decrease when a critical value was reached. However, this did not indicate membrane destruction, and the membrane voltage dropped to a specific level and oscillated around this level. Similar oscillations have been observed and described by other authors [13, 27]. These oscillations were interpreted as the generation (under constant current) of a variable-size pore [13, 14]. The third region of current densities (Fig. 4, curve *iii*) represents the current densities at which membrane destruction (irreversible breakdown) occurred—a rapid voltage increase is followed by a sudden drop to zero. The membrane under study was unable to withstand a current density of 233 nA cm^−2^, as demonstrated by the sudden disappearance of the bilayer voltage indicating membrane destruction.

**Fig. 5:**
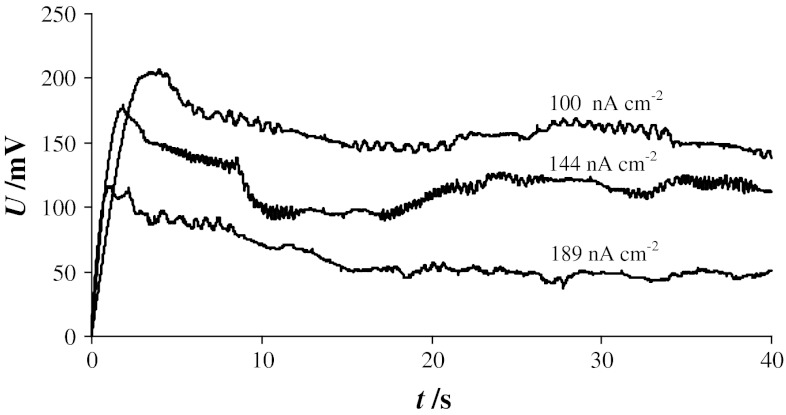
Chronopotentiometric curves for phosphatidylcholine–cholesterol bilayer (1:1 molar ratio) recorded at several constant current densities

Figure 5 depicts examples of chronopotentiometric curves recorded sequentially for one PC–Ch membrane (molar ratio 1:1) at current densities of 100, 144, and 189 nA cm^−2^. The curve shapes are very similar to curve *ii* in Fig. 4 and are characteristic of pore formation, that is, a rapid increase in membrane voltage followed by a sudden decrease after reaching a critical value. Again, rather than drop to the starting value of 0 mV, the voltage oscillates about an intermediate value, indicating that the bilayer is not destroyed.

The curves presented in Fig. 5 demonstrate the chronopotentiometric measurement sequence: After recording of the first curve, the measurements were paused for 30 s, and a second curve was recorded at higher current density. After a similar pause, the third curve was recorded. Although chronopotentiometry is relatively safe for membranes and enables recording of several chronopotentiometric curves for a single bilayer, it should not be utilized in experiments in which values are compared. Long-term electroporation significantly alters the lipid orientation around the pore rim (probably more than in pulsing electroporation), and the membrane “remembers” the pore for several minutes after resealing. As a side effect, subsequent electroporation is easier and the necessary transmembrane voltage is much lower.

When combining chronopotentiometric curves obtained for the same membrane at different current densities such as in Fig. 5, it is apparent that the speed at which changes in membrane voltage occur is dependent on the current density—higher current densities result in faster increases in membrane voltage. The same tendency was observed in all measurements and is in accordance with the literature [21]. Chronopotentiometric curves similar to curve *ii* in Fig. 4 and the curves in Fig. 5 are used to examine the process of pore formation, changes in pore size over time, and pore closing leading to complete membrane recovery. This type of chronopotentiometric curve may also be used to calculate the pore diameter [19].

In bilayers formed from pure phosphatidylcholine, effects indicating pore appearance were not observed on chronopotentiometric curves at current densities from 11 to 189 nA cm^−2^, while increases in current density beyond 189 nA cm^−2^ destroyed the PC membranes. In our opinion, this is due to a small amount of water present in the PC membranes which causes pores formed in these bilayers not to disappear but instead to greatly increase in size, leading to membrane destruction.

### Effect of Cholesterol on Capacitance and Resistance of the Membranes at Low Current Conditions

**Fig. 6:**
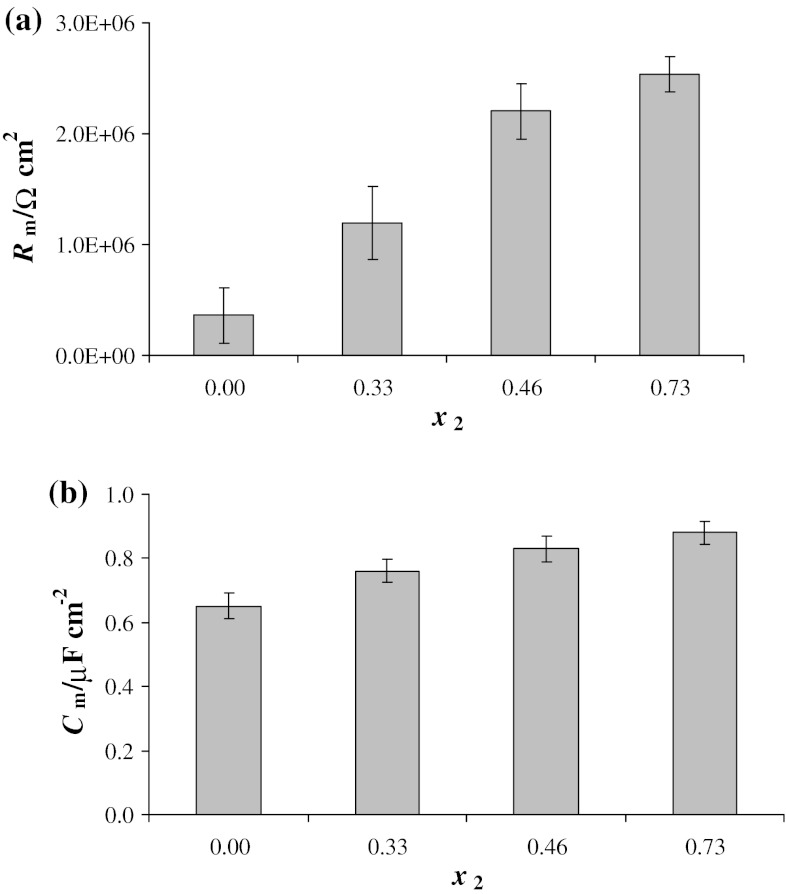
Dependence of resistance *R*_m_ (**a**) and capacitance *C*_m_ (**b**) of phosphatidylcholine–cholesterol membranes on the molar ratio of cholesterol. Each point represents the mean value (±S.D.) of results obtained from six bilayers

Chronopotentiometric curves recorded at low current density (such as curve *i* in Fig. 4) are very useful in calculating the electrical capacitance and the electrical resistance of bilayer lipid membranes. The composition dependence of the resistance and capacitance of a PC-Ch system are illustrated in Fig. 6 (the molar ratio values are represented by central values of the intervals). The values of *R*_m_ and *C*_m_ were derived by least squares fitting from *U* = *f*(*t*) curves obtained at a current density of 33 nA cm^−2^. Using Eq. (2) and the fact that in the absence of pores *R*_m_ obeys Ohm’s law, the resistance was calculated from the linear portion of the chronopotentiometric curves. The capacitance was calculated from the slopes of the curves using Eq. (3). Both parameters were normalized for bilayer area.

All membrane measurements yielded similar results, indicating good reproducibility of the electrical behavior. Although it is not possible to completely control the membrane formation process and every membrane is slightly different, the *R*_m_ and *C*_m_ values were consistent. The value of *R*_m_ obtained for a PC membrane was 3.64 ± 0.32 × 10^5^ Ω cm^2^, and the mean *C*_m_ value of a PC bilayer was 0.66 ± 0.04 μF cm^−2^, similar to values reported in the literature [28, 29]. When a passive RC circuit with electrical parameters corresponding to the typical BLMs formed in our laboratory was analyzed using electrochemical impedance spectroscopy, results similar to those obtained for pure phosphatidylcholine membranes at low current density values (no electroporation) were obtained [17, 30]. Impedance spectroscopy is a noninvasive technique capable of accurately measuring electrical parameters [31–33].

### Effect of Cholesterol on Pore Conductance and Diameter

**Fig. 7:**
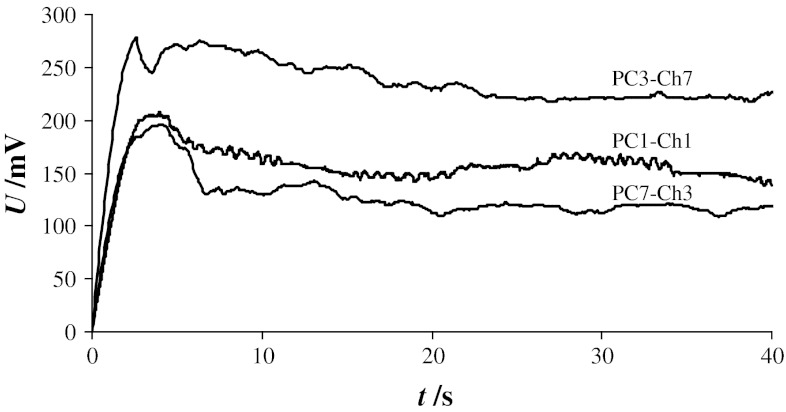
Chronopotentiometric characteristics of bilayer lipid membranes containing various molar ratios of phosphatidylcholine to cholesterol (current density—100 nA cm^−2^)

Pore conductance and diameter calculation methods are presented in the Theory section (Subsect. 2.2). The pore conductance was calculated according to Eq. (8) from chronopotentiometric curves recorded at a current density of 100 nA cm^−2^ (the curves appear in Fig. 7). In order to examine pore evolution and reversibility in the membrane structure, the calculations were performed at three points on each chronopotentiometric curve. Points were chosen arbitrarily in order to characterize pore conductance at different instances in the pore lifetime.

Our experiments indicate that the pore conductance was affected by the sterol content of the membranes. The average level of *G*_p_ fluctuations decreased with increasing cholesterol content. The *G*_p_ values were 91.5 ± 18.3 nS for a PC7–Ch3 membrane, 40.4 ± 8.3 nS for a PC1–Ch1 membrane, and 13.2 ± 2.4 nS for a PC3–Ch7 membrane. The mean conductance provides information about the mean pore diameter. The mean pore diameters calculated using Eq. (9) were 8.9, 5.9, and 3.4 nm for PC7–Ch3, PC1–Ch1, and PC3–Ch7 bilayers.

**Fig. 8:**
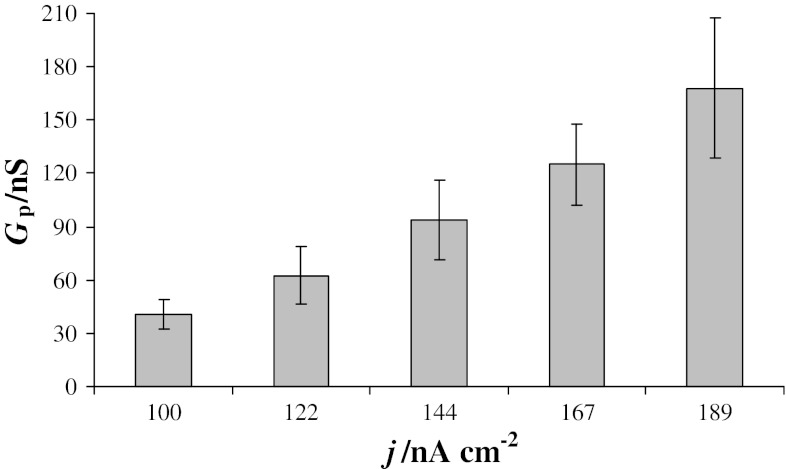
Dependence of the pore conductance *G*_P_ calculated for phosphatidylcholine–cholesterol membranes (1:1 molar ratio) on the current density *j*. Each point represents the mean value (±S.D.) of results obtained from six bilayer lipid membranes

The data presented in Fig. 8 demonstrate that the conductance of fluctuating pores is dependent on the current flow through the bilayer. Higher current densities cause an increase in pore conductance. The average conductances of PC1–Ch1 membranes calculated from chronopotentiometric curves recorded at current densities of 100, 122, 144, 167, and 189 nA cm^−2^ were 40.4 ± 8.3, 62.4 ± 16.1, 93.6 ± 22.6, 125 ± 27, and 168 ± 39 nS.

**Fig. 9:**
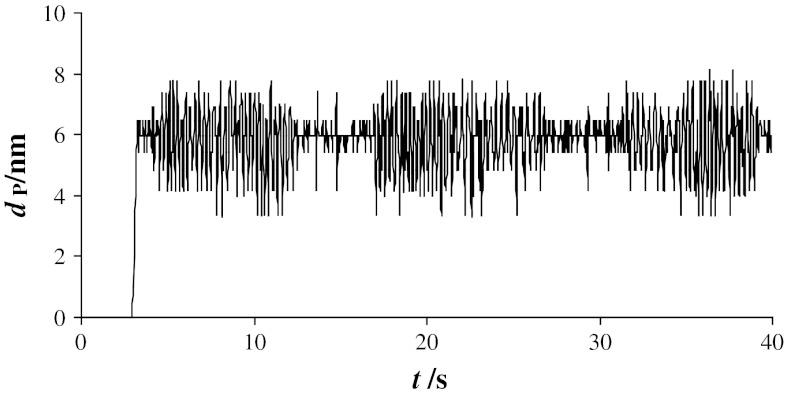
Dependence of pore diameter *d*_P_ on time *t* in membrane formed from phosphatidylcholine and cholesterol (1:1 molar ratio). *Current density* 100 nA cm^−2^

The mean diameters of pores generated in the membranes detailed above were 5.9, 7.6, 8.5, 9.9, and 12.2 nm. All pores exhibit dimensional fluctuation with time, and the time dependence of pore diameter illustrated in Fig. 9 provides an indication of pore stability.

### Effect of Cholesterol on Breakdown Voltage

Breakdown voltages were determined using chronopotentiometric curves obtained at a current density of 100 nA cm^−2^ for several lipid compositions (Fig. 7). The highest membrane voltage recorded just before the collapse of the curve was interpreted as the breakdown voltage at which a stable pore may be formed in a membrane. Voltage fluctuations in these curves were due to changes in pore size.

The current density for reversible breakdown is mainly dependent upon the materials used for membrane preparation. As expected, incorporation of cholesterol increased the breakdown voltage. Using our apparatus and experimental conditions, the *U*_C_ value for a PC7–Ch3 membrane was 190 ± 28 mV, compared to 205 ± 35 mV for a PC1–Ch1 membrane and 280 ± 31 mV for a PC3–Ch7 membrane.

## Discussion

This manuscript presents a chronopotentiometric study of BLMs subjected to constant current electroporation to determine the influence of cholesterol on the electrical properties of PC membranes, their sensitivity to electric fields, and membrane characteristics during long-term electroporation. This is an important topic, especially since cholesterol plays a major role in plasma membrane properties and cellular processes. One topic that has recently been the subject of intense interest in many research groups is the role of membrane lipid composition (particularly with regard to cholesterol content) in neurodegenerative disorders such as Alzheimer’s disease [34, 35].

### Capacitance and Resistance of Membranes (Calculated from Low Current Chronopotentiometric Curves)

Capacitance is considered the best tool for probing the stability and quality of formation of lipid bilayers [28]. The resistance of a bilayer may vary by at least one order of magnitude due to impurities, border leakage at the membrane support, the appearance of lipid “crystals” at the periphery of the BLM, or the method used to introduce the lipid solution (if the forming solution is introduced using a microsyringe instead of a brush, bilayer irreproducibility can be minimized). However, the resistance of an individual membrane is usually constant until a short time before the membrane is disrupted, and any changes in resistance due to addition of ions, proteins, drugs, etc., may be determined with relatively high accuracy [20].

The composition dependence of membrane resistance described in this paper is nonlinear (Fig. 6a), which could be caused by the formation of bonds in the membrane. Hydrogen bonding between lipid heads and between chains should be taken into consideration when interpreting lipid–lipid interactions in the membrane. At the membrane/aqueous solution interface, water molecules may take part in a network of hydrogen bonds stretching between the lipids in the bilayer [36].

Lipid–lipid interactions are stronger in lipids whose amphiphilic phosphocholine heads are hydrated [37]. “Polar pores,” sometimes called defects, may be formed in synthetic lipid bilayers as the result of even small differences in osmotic pressure [38]. It should be emphasized that these short-lived defects are not proper electropores. With a PC membrane, the hydrophilic heads of the lipids are oriented almost parallel to the bilayer plane [39]. The phosphatidylcholine head diameter is much larger than the diameter of the thin hydrophobic chain, which is why separation distances are mainly determined by the head group diameter. Some head groups may face the interior of the membrane surrounded by their associated water molecules. The hydrocarbon tails have extensive freedom to move, and there is sufficient space between them to accommodate water molecules. The presence of water molecules in PC bilayers (or in bilayers predominantly composed of PC) increases the electrical conductivity of the bilayer. The arrangement of water molecules in the hydrophobic portion of the membrane is random and irreproducible.

The presence of cholesterol in the bilayer does not modify the orientation of the lipid heads because the cholesterol molecules are oriented parallel to the phosphatidylcholine chains and their hydroxyl groups are located at the level of the lipid carbonyl groups [23]. Hydrocarbon chain mobility is markedly reduced by the presence of cholesterol (the so-called condensation effect), but it has no effect on the terminal methyl group. Because cholesterol is shorter but more voluminous, the phosphatidylcholine chains are folded back with their methyl ends filling the void appearing below the cholesterol molecule [40]. The total membrane thickness is thereby reduced, as well as the amount of water in the bilayer. In bilayers containing mostly cholesterol, the intermolecular distances are determined by the hydrophobic diameter of the molecule, and conditions do not favor the presence of a significant number of water molecules in the hydrophobic region.

The above phenomenon is reflected in the dependence of membrane capacitance and resistance on the molar ratio of PC–Ch (Fig. 6a, b). A slight scattering of the experimental values is observed in *C*_m_ measurements due to the slight sensitivity of capacitance to the presence of a small amount of water in the lipid layer. In contrast, the resistance curves exhibit a large amount of scatter in membranes containing mostly PC. The scatter is much smaller for membranes in which cholesterol is the predominant component. The low resistance of membranes formed by us using a painting technique indicates that the membranes are capable of ion transport, making it reasonable to assume that a small amount of water is present in each membrane.

The effect of cholesterol on phosphatidylcholine membrane capacitance and resistance has been extensively studied [41, 42]. Our present results confirm the earlier observations in demonstrating that cholesterol induces changes in the PC bilayer including reduced mobility of the alkyl chains, greater membrane order and stiffness, and lower permeability.

### Pore Conductance, Pore Diameter, and Breakdown Voltage (Calculated from Chronopotentiometric Curves Recorded at High Currents)

Transient defects and pores in the membrane structure affect its conductance, causing measurement fluctuations. Capacitance and conductance changes in planar lipid membranes are typically measured using voltage clamp techniques at low potentials [43]. The fluctuations are more pronounced under electric fields that are sufficiently strong to cause electroporation. Pore appearance is preceded by lipid reorganization, resulting in transient increases in the membrane permeability to ions. In response, current fluctuations are observed prior to the irreversible breakdown of planar lipid bilayers [44]. Following electroporation, it is unlikely that a pore will maintain fixed dimensions, and pore fluctuations are theoretically predicted. Since electroporation under voltage clamp conditions results in very fast pore expansion leading to rapid membrane breakdown, experimental studies of pore dynamics in the voltage clamp mode require the application of very short pulses to prevent membrane destruction [45, 46]. The experiments reported in Ref. [46] yielded estimates of the typical lifetime of an electropore created under voltage clamp conditions (250 mV) as 3 ms. Conductance fluctuations recorded in these experiments were attributed to pore dynamics. However, the voltage was clamped above the breakdown voltage, and because of the high potential, the appearance of multiple pores is almost certain. The combined dynamics of several pores may have accounted for the fluctuations and hindered the observation of single pore dynamics [47, 48].

Because of slow charge accumulation, exposure of the planar lipid bilayer to a constant current does not lead to rapid breakdown. When the first pore appears, the transmembrane voltage decreases, preventing subsequent pore formation and hypothetically enabling formation of a single pore. The fluctuations observed in current clamp experiments are caused by the opening and closing of a single pore [14, 19, 49–51]. The natural electropore fluctuations are enhanced by a negative feedback inherent to the current clamp electroporation method. The feedback results from interaction between the pore surface and the transmembrane voltage. As a consequence of pore expansion, the membrane resistance decreases and the voltage across the bilayer is reduced. This prevents a further increase in size of the electropore, which instead generally starts to shrink, increasing the transmembrane voltage. This chain of events accounts for the observed pore stability apart from slight fluctuations. The pore lifetime can be as long as several hours [12].

The voltage oscillations recorded in our experiments at intermediate current densities for BLMs with varying lipid composition were regarded as due to the generation (under constant current density) of cyclically opening and closing pores [19]. The decrease in *G*_p_ with increasing cholesterol content reflects the condensing effect of cholesterol on the phosphatidylcholine bilayer.

The conductance of the fluctuating pores depends on the current flow through the bilayer: Higher current density causes an increase in pore conductance. The results presented in this paper (Fig. 8) demonstrate an almost linear relationship between pore conductance and current density, suggesting that all of these values may be related to a single oscillating electropore or to pores appearing and disappearing one at a time [19]. The higher pore conductance observed at higher current density indicates a larger pore diameter. BLMs modified by the incorporation of cholesterol demonstrate greater membrane stability, and pores may achieve larger diameters and carry greater currents without causing membrane destruction.

A conductance analysis of electroporated membranes [14, 19] indicates that single pores have average diameters ranging from 0.9 nm (at low current and high ionic strength) to 10.56 nm (at high current and low ionic strength). Interestingly, the pore size is comparable to the membrane thickness (~7 nm) and the individual molecular size. Molecules such as cholesterol that have small polar head (a single OH group) and a bulky hydrophobic portion inhibit the formation of pores and lower the conductance of the bilayer [29, 39]. Similar effects would be expected and have been reported [52] after the addition of molecules such as dolichyl phosphate to lipid bilayers. Conversely, lipid molecules with smaller non-polar volumes or relatively large polar heads would be expected to pack more favorably into the curved region of the pore. This would decrease the surface free energy in the curved surface and hence decrease the critical pore energy and critical pore radius. For example, lysolecithin (a form of phosphatidylcholine containing the same head group but only one hydrocarbon tail) would favor pore formation [29].

The breakdown voltage *U*_C_ is one of the most important properties of lipid bilayers to consider in biomedical and biotechnological applications of electroporation [28]. It differs between natural and artificial lipid membranes and is also dependent on the analysis method when applied to similar bilayers. Its value is specific for membrane composition, dimension, and shape. The influence of organic solvents, temperature, and electrolyte composition on the *U*_c_ voltage value is described in the literature [53–55]. In our experiments, we tested the influence of cholesterol content on the breakdown voltage. The increase in breakdown voltage value with increasing amounts of Ch in the membrane reflects the condensing effect of cholesterol on the phosphatidylcholine bilayer, in contrast to the effects caused by other molecules with hydroxyl head groups such as polyprenol [56] or *α*-tocopherol [13]. There are discrepancies in the breakdown voltage values reported in the literature, particularly in data for membranes containing cholesterol. Needham et al. [57] reported that *U*_C_ increased with increasing Ch content in vesicles, while Genco et al. [27] found almost no difference in *U*_C_ between pure and cholesterol-modified PC membranes.

## Conclusions

The application of chronopotentiometry to the study of membrane phenomena provides a great deal of useful membrane parameters including breakdown voltage, membrane capacitance and resistance at various current densities, and pore conductance and diameter, several of which are affected by biologically active substances. The proposed method is also rapid, which is particularly important when examining time-dependent phenomena in bilayers.

## References

[1] Tomov TC (1995). Quantitative dependence of electroporation on the pulse parameters. Bioelectrochemistry and Bioenergetics.

[2] Valero A, Post JN, van Nieuwkasteele JW, Ter Braak PM, Kruijer W, Van Den Berg A (2008). Gene transfer and protein dynamics in stem cells using single cell electroporation in a microfluidic device. Lab on a Chip.

[3] Granot Y, Rubinsky B (2008). Mass transfer model for drug delivery in tissue cells with reversible electroporation. International Journal of Heat and Mass Transfer.

[4] Wells DJ (2004). Gene therapy progress and prospects: Electroporation and other physical methods. Gene Therapy.

[5] Golberg A, Fischer J, Rubinsky B, Rubinsky B (2010). The use of irreversible electroporation in food preservation. irreversible electroporation.

[6] Puc M, Corovic S, Flisar K, Petkovsek M, Nastran J, Miklavcic D (2004). Techniques of signal generation required for electropermeabilisation. Survey of electropermeabilisation devices. Bioelectrochemistry.

[7] Macek Lebar A, Sersa G, Kranjc S, Grošelj A, Miklavčič D (2002). Optimisation of pulse parameters in vitro for in vivo electrochemotherapy. Anticancer Research.

[8] Freeman SA, Wang MA, Weaver JC (1994). Theory of electroporation of planar bilayer membranes: Predictions of the aqueous area, change in capacitance, and pore–pore separation. Biophysical Journal.

[9] Jain MK (1972). The bimolecular lipid membrane.

[10] Chizmadzhev YA, Guidelli R (1992). Structural rearrangements in lipid bilayer membranes. Electrified interfaces in physics, chemistry and biology, NATO ASI Ser C.

[11] Robello M, Gliozzi A (1989). Conductance transition induced by an electric field in lipid bilayers. Biochimica et Biophysica Acta.

[12] Kotulska M, Koronkiewicz S, Kalinowski S (2004). Self-similar processes and flicker noise from a fluctuating nanopore in a lipid membrane. Physical Review E.

[13] Koronkiewicz S, Kalinowski S, Bryl K (2001). Changes of structural and dynamic properties of model lipid membranes induced by alfa-tocopherol: implication to the membrane stabilization under external electric field. Biochimica et Biophysica Acta.

[14] Koronkiewicz S, Kalinowski S, Bryl K (2002). Programmable chronopotentiometry as a tool for the study of electroporation and resealing of pores in bilayer lipid membranes. Biochimica et Biophysica Acta.

[15] Naumowicz M, Kotynska J, Petelska AD, Figaszewski ZA (2006). Impedance analysis of phosphatidylcholine modified with valinomycin. European Biophysics Journal.

[16] Naumowicz M, Figaszewski ZA (2003). Impedance analysis of phosphatidylcholine membranes modified with gramicidin D. Bioelectrochemistry.

[17] Naumowicz M, Figaszewski ZA (2005). Impedance analysis of lipid domains in phosphatidylcholine bilayer membranes containing ergosterol. Biophysical Journal.

[18] Naumowicz M, Figaszewski ZA (2009). Impedance spectroscopic investigation of the interaction between phosphatidylethanolamine and α-tocopherol in bilayer membranes. Electrochimica Acta.

[19] Kalinowski S, Ibron G, Bryl K, Figaszewski Z (1998). Chronopotentiometric studies of electroporation of bilayer lipid membranes. Biochimica et Biophysica Acta.

[20] Tien HT (1974). Bilayer lipid membrane: Theory and practice.

[21] Koronkiewicz S, Kalinowski S (2004). Influence of cholesterol on electroporation of bilayer lipid membrane: Chronopotentiometric studies. Biochimica et Biophysica Acta.

[22] Benz R, Fröhlich O, Läuger P, Montal M (1975). Electrical capacity of black lipid films and of lipid bilayers made from monolayers. Biochimica et Biophysica Acta.

[23] Karolins C, Coster HGL, Chilcott TC, Barrow KD (1998). Differential effects of cholesterol and oxidized-cholesterol in egg lecithin bilayers. Biochimica et Biophysica Acta.

[24] Bronsztejn IN, Siemiendiajew KA (1996). Mathematics. The encyclopedic handbook.

[25] Figaszewski Z (1982). System for measuring separate impedance characteristics with a three or four-electrode potentiostat. Journal of Electroanalytical Chemistry.

[26] Figaszewski Z, Koczorowski Z, Geblewicz G (1982). System for electrochemical studies with a four-electrode potentiostat. Journal of Electroanalytical Chemistry.

[27] Genco I, Gliozzi A, Relini A, Robello M, Scalas E (1993). Electroporation in symmetric and asymmetric membranes. Biochimica et Biophysica Acta.

[28] Pavlin M, Kotnik T, Miklavčič D, Kramar P, Macek-Lebar A, Leitmannova Liu A (2008). Electroporation of planar lipid bilayers and membranes. Advances in planar lipid bilayers and liposomes.

[29] Coster HGL, Ottova-Leitmannova A, Tien HT (2003). Dielectric and electrical properties of lipid bilayers in relation to their structure. Planar lipid bilayers (BLMs) and their applications.

[30] Naumowicz M, Petelska AD, Figaszewski ZA (2005). Impedance analysis of a phosphatidylcholine-phosphatidylethanolamine system in bilayer lipid membrane. Bioelectrochemistry and Bioenergetics.

[31] Gal M, Hives J, Sokolova R, Hromadova M, Kolivoska V, Pospisil L (2009). Impedance study of hypoxic cells radiosensitizer etanidazole radical anion in water. Collection of Czechoslovak Chemical Communications.

[32] Hromadova M, Gal M, Bulickova J, Sokolova R, Fanelli N (2008). Electrochemical impedance of nitrogen fixation mediated by fullerene-cyclodextrin complex. Electrochimica Acta.

[33] Gal MF, Hives J, Benova M, Galova M (2007). Electrochemical impedance and conductivity measurements in a heterogeneous Fe powder particle-electrolyte system with or without electrochemical reaction. Journal of Applied Electrochemistry.

[34] Biondi E (2011). Prescription of lipophilic statins to Alzheimer’s disease patients: Some controversies to consider. Neurological Sciences.

[35] Canevari L, Clark JB (2007). Alzheimer’s disease and cholesterol: the fat connection. Neurochemical Research.

[36] Boggs JM (1987). Lipid intermolecular hydrogen bonding: Influence on structural organization and membrane function. Biochimica et Biophysica Acta.

[37] Slater SJ, Ho C, Taddeo FJ, Kelly MB, Stubbs CD (1993). Contribution of hydrogen bonding to lipid–lipid interactions in membranes and the role of lipid order: Effects of cholesterol, increased phospholipids unsaturation, and ethanol. Biochemistry.

[38] Taupin C, Dvolaitzky M, Saterey C (1975). Osmotic pressure induced pores in phospholipid vesicles. Biochemistry.

[39] McIntosh T (1978). The effect of cholesterol on the structure of phosphatidylcholine bilayers. Biochimica et Biophysica Acta.

[40] Bhattacharya S, Haldar S (2000). Role of lipid headgroup and hydrocarbon chain-backbone linkage between cholesterol and lipids in bilayer membranes. Biochimica et Biophysica Acta.

[41] Brosseau CL, Bin X, Roscoe SG, Lipkowski J (2008). Electrochemical and PM-IRRAS characterization of DMPC + cholesterol bilayers prepared using Langmuir–Blodgett/Langmuir–Schaefer deposition. Journal of Electroanalytical Chemistry.

[42] Watala C, Drapeza A, Loban V, Asztemborska M, Shcharbin D (2009). Effect of acetylsalicylic acid on the current-voltage characteristics of planar lipid membranes. Biophysical Chemistry.

[43] Chanturiya AN (1990). Detection of transient capacitance increase associated with channel formation in lipid bilayers. Biochimica et Biophysica Acta.

[44] Abidor IG, Arakelyan VB, Chernomordik LV, Chizmadzhev YA, Pastushenko VF, Tarasevich MR (1979). Electric breakdown of bilayer membranes: I. The main experimental facts and their qualitative discussion. Bioelectrochemistry and Bioenergetics.

[45] Antonov VF, Petrov VV, Molnar AA, Prevoditelev DA, Ivanov AS (1980). The appearance of single-ion channels in unmodified lipid bilayer membranes at the phase transition temperature. Nature.

[46] Melikov KC, Frolov VA, Shcherbakov A, Samsonov AV, Chizmadzhev YA, Chernomordik LV (2001). Voltage-induced nonconductive pre-pores and metastable single pores in unmodified planar lipid bilayer. Biophysical Journal.

[47] Diederich A, Bähr G, Winterhalter M (1998). Influence of surface charges on the rupture of black lipid membranes. Physical Review E.

[48] Sharma V, Uma Maheswari K, Murphy JC, Tung L (1996). Poloxamer 188 decreases susceptibility of artificial lipid membranes to electroporation. Biophysical Journal.

[49] Kalinowski S, Figaszewski Z (1995). A four-electrode system for measurement of bilayer lipid membrane capacitance. Measurement Science & Technology.

[50] Kalinowski S, Figaszewski Z (1995). A four-electrode potentiostat-galvanostat for studies of bilayer lipid membranes. Measurement Science & Technology.

[51] Kotulska M (2007). Natural fluctuations of an electropore show fractional Lévy stable motion. Biophysical Journal.

[52] Janas T, Tien HT (1988). Influence of dolichyl phosphate on permeability and stability of bilayer lipid membranes. Biochimica et Biophysica Acta.

[53] Benz R, Janko K (1976). Voltage-induced capacitance relaxation of lipid bilayer membranes; effects on membrane composition. Biochimica et Biophysica Acta.

[54] Benz R, Beckers F, Zimmermann U (1979). Reversible electrical breakdown of lipid bilayer membranes: A charge-pulse relaxation study. Journal of Membrane Biology.

[55] Wilhelm C, Winterhalter M, Zimmermann U, Benz R (1993). Kinetics o pore size during irreversible electrical breakdown of lipid bilayer membranes. Biophysical Journal.

[56] Janas T, Walińska K, Janas T (1998). Electroporation of polyprenol-phosphatidylcholine bilayer lipid membranes. Bioelectrochemistry and Bioenergetics.

[57] Needham D, Nelson P (1997). Dynamically stabilized pores in bilayer membranes. Biophysical Journal.

